# Anticipated discrimination and suicidal behaviors among Black gay and bisexual male adolescents: the moderating role of developmental assets

**DOI:** 10.1186/s13034-026-01128-y

**Published:** 2026-06-29

**Authors:** Donte T. Boyd, Arielle H. Sheftall

**Affiliations:** 1 College of Social Work, The Ohio State University, 1047 College RD, Columbus, OH 43210 USA; 2 Department of Psychiatry, University of Rochester Medical Center, University of Rochester, Rochester, NY 14609 USA

**Keywords:** Suicidal behaviors, Positive identity, Social competencies, Positive values, Anticipated discrimination, Black gay and bisexual male adolescents

## Abstract

**Background:**

Black gay and bisexual male adolescents (BGBMA) face disproportionately high rates of suicidal behaviors, shaped by intersecting racial and sexual minority stressors. Anticipated discrimination—a future-oriented minority stressor—may be an important yet understudied correlate of suicidality in this population. Guided by the Developmental Assets Framework, this study examined whether internal developmental assets (positive identity, positive values, social competencies) mitigate the association between anticipated discrimination and suicidal behaviors among BGBMA.

**Methods:**

Data were drawn from 384 BGBMA aged 14–17 years residing in three Midwestern U.S. cities who completed an anonymous online Qualtrics survey between December 2023 and January 2024. Suicidal behaviors were assessed using a self-report adaptation of the Columbia–Suicide Severity Rating Scale (C-SSRS). Anticipated discrimination was measured with a nine-item subscale of the Intersectional Discrimination Index, and internal developmental assets were assessed with validated scales of positive identity, positive values, and social competencies. Linear regression models tested main effects of anticipated discrimination and internal assets on suicidal behaviors, followed by moderation analyses examining interaction terms between anticipated discrimination and each asset.

**Results:**

Higher anticipated discrimination was significantly associated with greater suicidal behaviors (b = 0.46, *p* = 0.001). Positive identity (b = − 0.56, *p* < 0.001) and social competencies (b = − 0.78, *p* < 0.001) were each associated with lower suicidal behaviors. Two significant moderation effects emerged. Positive identity buffered the association between anticipated discrimination and suicidal behaviors (interaction b = − 0.23, *p* = 0.011); at high levels of positive identity, anticipated discrimination was no longer significantly associated with suicidal behaviors. In contrast, social competencies amplified the discrimination–suicidality association (interaction b = 0.49, *p* = 0.015), such that anticipated discrimination was most strongly related to suicidal behaviors at high levels of social competencies.

**Conclusions:**

Anticipated discrimination is a robust correlate of suicidal behaviors among BGBMA. Internal developmental assets do not function uniformly: positive identity appears protective, whereas higher social competencies may confer vulnerability under conditions of anticipated discrimination. Findings underscore the need for developmentally and culturally grounded suicide prevention strategies that both strengthen identity-related assets and address discrimination-related stress in Black gay and bisexual male adolescents.

## Introduction

Suicide remains a major public health concern, with more than 6000 youth and young adults ages 10 to 24 years dying by suicide in 2023 [1]. Epidemiological data consistently show that males have higher suicide mortality rates than females, and LGBTQ+ youth represent a group at particularly elevated risk [2–4]. LGBTQ+ individuals are approximately three times more likely to consider suicide and up to five times more likely to attempt suicide compared to their heterosexual peers [5]. According to the 2023 Youth Risk Behavior Surveillance Study, 40.6% of youth identifying as LGBTQ+ seriously considered suicide, and one in five reported a suicide attempt [2]. These disparities are also reflected in contemporary LGBTQ+ youth data; for example, The Trevor Project’s 2024 U.S. National Survey found that 39% of LGBTQ+ young people seriously considered attempting suicide in the past year, and prior analyses indicate that LGBTQ+ youth are more than four times as likely to attempt suicide as their peers.

Within this broader population, Black gay and bisexual male adolescents and young adults (BGBMA; ages 14–24) experience particularly elevated levels of suicide risk. Recent evidence indicates that 42% of BGBMA report a lifetime nonfatal suicide attempt, 39% report an aborted attempt, and 33% report an interrupted attempt. Additionally, 40% report engagement in preparatory suicidal behaviors (e.g., writing a suicide note) [5, 6]. These findings highlight the high prevalence and complexity of suicidal behaviors within this population and underscore the need to examine mechanisms that contribute to suicide risk among Black sexual minority youth across developmental stages.

Among sexual minority youth, Black gay and bisexual male adolescents experience disproportionately elevated risk for suicidal ideation and behaviors [7]. Recent work by Boyd et al. draws on intersectionality and highlights how the convergence of racialized and sexual minority statuses exposes BGBMA to layered forms of racism, homophobia, and HIV-related stigma, which jointly elevate depression and suicidality [8]. In a national sample of BGBMA, Boyd et al. demonstrated that suicidal ideation, planning, and attempts were highly concentrated among youth reporting low familial support and poor familial communication [9]. These findings underscore the impact of interpersonal contexts on minority stress and its mental health consequences.

Complementary analyses of BGBMA (14–24 years) demonstrate that internal developmental assets—defined as the 20 personal skills, values, and self-perceptions that help young people make positive choices, thrive, and take responsibility for their lives—mitigate suicidal behaviors even when multiple minority stressors are present [6, 10]. These assets include constructs such as positive identity (e.g., self-esteem and sense of purpose), social competencies (e.g., interpersonal and decision-making skills), and positive values (e.g., integrity and responsibility) [6]. These findings align with Mustanski and Liu’s longitudinal evidence that early exposure to minority stress escalates depressive symptoms and suicidality and correspond with Burton et al.’s work demonstrating that racism- and homophobia-related victimization exert distinct and deleterious effects on the mental health of Black sexual minority boys [10–12].

Despite these contributions, most studies remain cross-sectional and emphasize distal outcomes (e.g., reported suicide attempts) rather than upstream processes such as youths anticipating and internalizing potential discrimination across social settings. Minority stress theory provides a useful framework for understanding these patterns by distinguishing between distal stressors (e.g., experiences of discrimination) and proximal stressors (e.g., internalized stigma and negative expectations about future mistreatment) [13–16]. Proximal stress processes, including anticipatory vigilance and expectations of discrimination, are thought to increase psychological burden through heightened stress appraisal, anxiety, and hopelessness, thereby elevating risk for adverse mental health outcomes, including suicidality [13–16]. Existing research demonstrates that anticipated discrimination consistently predicts elevated depression, anxiety, and suicidal ideation among racially and sexually minoritized populations [17, 18]. Studies of Black gay and bisexual men demonstrate how intersectional stigma—including racial, sexual, and HIV-related stigma—generates psychological distress and reduces engagement with preventive and mental health services [19–21]. Research on internalized and anticipated stigma further indicates cumulative effects on depressive symptoms and suicidality, particularly among individuals facing socioeconomic marginalization [22, 23].

Anticipated discrimination is a critical, yet understudied, minority stressor that may help explain how intersectional stigma becomes embodied as depression, hopelessness, and suicidality among BGBMA. Despite the converging lines of evidence, the literature has not adequately examined how anticipated discrimination shapes mental health trajectories among Black sexual minority adolescents—a group simultaneously navigating identity development and heightened vulnerability to stigma-related stress. Emerging work documenting Black sexual minority youths’ experiences of racism and heterosexism [24] signals the urgency of understanding these dynamics earlier in the life course, where cumulative exposure may influence long-term risk. Culturally grounded, developmentally tailored interventions fostering resilience, community connection, and mental health literacy are crucial to mitigating the adverse effects of anticipated discrimination during adolescence [19, 25, 26].

### Developmental assets framework and minority stress theory

The Developmental Assets Framework (DAF) offers a strengths-based model for understanding how internal and external resources support healthy development, particularly during adolescence—a developmental period marked by increasing autonomy, identity exploration, and heightened vulnerability to social stressors [27–29]. According to DAF, youth thrive when they can access internal assets (e.g., positive identity, coping skills, and decision-making competence) alongside external assets (e.g., supportive family relationships, caring adults, and affirming community environments). Specifically, positive identity—a core internal asset—reflects a young person’s sense of self-worth, purpose, and positive self-regard, including confidence in one’s future and pride in one’s identity. Among Black gay and bisexual male adolescents (BGBMA), these assets may serve as critical protective resources in the context of intersecting racial and sexual minority stressors [27–31]. Empirical evidence indicates that internal assets, such as positive identity and coping skills, are associated with lower suicide risk even in the presence of racism, homophobia, and HIV-related stigma, while external assets—including family support and affirming social networks—further reduce the likelihood that minority stress translates into psychological distress and suicidal behaviors [6–10, 32, 33]. Collectively, DAF provides a robust framework for conceptualizing how strengths-based protective systems can promote resilience among Black sexual minority youth.

To more fully account for the stress processes shaping these outcomes, the present study is also informed by minority stress theory, which provides a complementary framework for understanding how stigma-related stressors influence mental health among sexual minority populations. Minority stress theory distinguishes between external experiences of stigma and discrimination and internal stress processes, such as negative expectations, vigilance, and anticipation of future mistreatment [13–16]. For BGBMA, anticipated discrimination may represent a key minority stress process through which expectations of rejection or unfair treatment rooted in intersecting racial and sexual identities shape psychological distress and suicide risk. Emerging evidence suggests that anticipated discrimination and related cognitive processes are associated with increased psychological burden, including heightened stress appraisal, anxiety, and poorer overall well-being.^¹³⁻¹⁵^ These anticipatory processes may, in turn, elevate vulnerability to suicidal behaviors.

Integrating these frameworks provides a more comprehensive understanding of both risk and resilience. Minority stress theory helps explain why anticipated discrimination may contribute to psychological vulnerability, whereas DAF identifies the internal strengths that may shape adolescents’ responses to these stressors. Developmental assets may buffer the association between anticipated discrimination and suicide risk; however, their protective effects may vary depending on the specific asset and outcome examined [15, 16]. In this context, internal developmental assets may function not only as buffers but also, under certain conditions, as amplifiers of stress-related outcomes. This integrated perspective is particularly relevant for BGBMA, whose developmental experiences are shaped by layered systems of marginalization and resilience [6, 10]. Despite this alignment, limited research has examined whether strengths-based assets shape the association between anticipated discrimination and suicidal behaviors among Black sexual minority adolescents.

### Current study

**Fig. 1:**
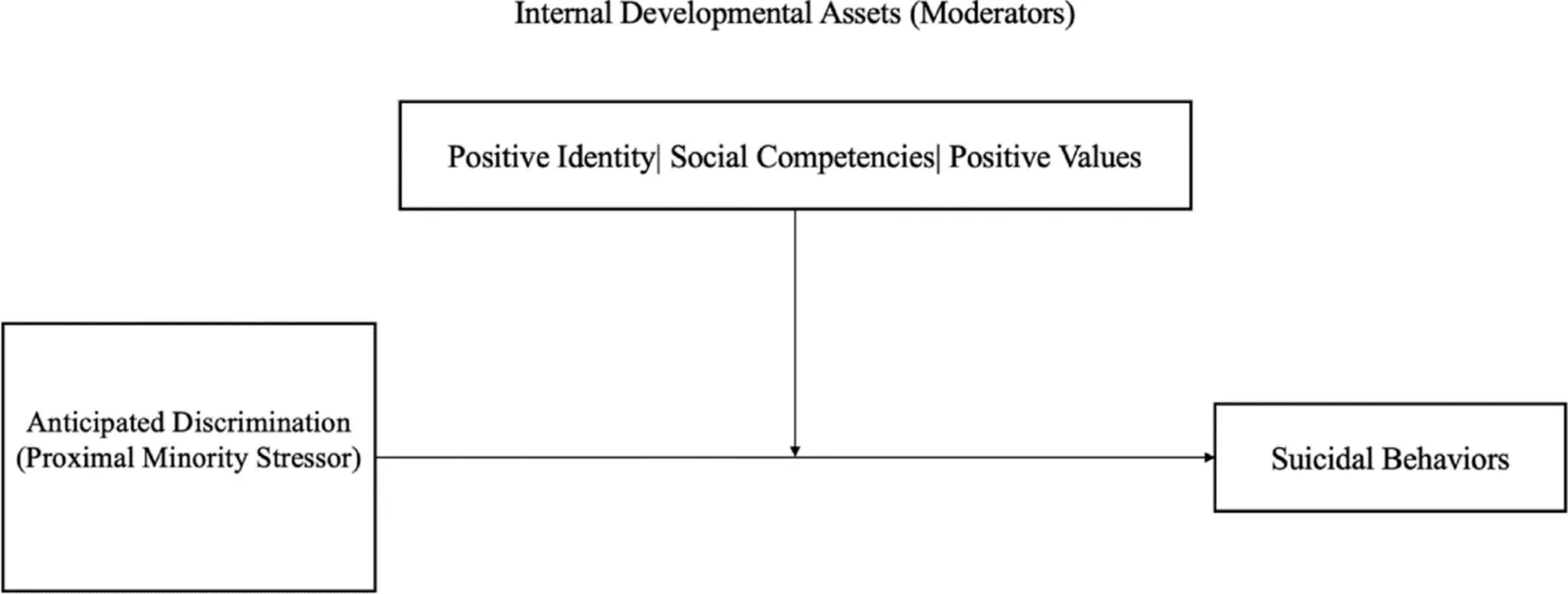
Conceptual model integrating minority stress and developmental assets frameworks for suicidal behaviors among BGBMA. The model illustrates the hypothesized association between anticipated discrimination and suicidal behaviors among Black gay and bisexual male adolescents and young adults. Internal developmental assets, including positive identity, social competencies, and positive values, are derived from the Developmental Assets Framework and are modeled as moderators that may influence the strength of this association

Drawing on the Developmental Assets Framework and minority stress theory, the present study examines whether positive internal assets buffer the association between anticipated discrimination and suicidal behaviors among BGBMA. Figure 1 presents the conceptual model guiding this study, illustrating the proposed relationships among anticipated discrimination, developmental assets, and suicidal behaviors in this population. The following research questions examined include:

1. How is anticipated discrimination associated with suicidal behaviors among BGBMA ages 14 to 17?
2. Do positive developmental assets (e.g., social competencies) moderate the relationship between anticipated discrimination and suicidal behaviors among BGBMA? If so, which assets are more protective?

We propose three hypotheses: (1) within BGBMA, higher levels of anticipated discrimination will be positively associated with suicidal behaviors; (2) higher levels of social competencies, positive values, and positive identity will be negatively associated with suicidal behaviors; and (3) social competencies, positive values, and positive identity will moderate the relationship between anticipated discrimination and suicidal behaviors.

## Methods

### Study procedures and recruitment

This study draws on data from a larger online survey examining developmental assets and health outcomes among Black sexual minority youth (ages 14–24; *N* = 583) residing in Midwestern U.S. cities [6, 13]. A total of 800 participants initially completed the survey; cases were excluded due to incomplete responses, failed attention checks, or missing data on key study variables, resulting in the final analytic sample of 583 participants. The present study focuses on the adolescent subsample (ages 14–17), yielding a final analytic sample of *N* = 384.

An anonymous link to the survey, generated by Qualtrics software, was embedded in a recruitment flyer [6, 13]. The research team promoted the survey through paid advertisements on social media platforms, including Facebook and X (formerly Twitter). Additionally, flyers were distributed through local community-based organizations and schools, with community health workers sharing them with eligible clients. Recruitment occurred between December 1, 2023, and January 31, 2024. All participants were required to provide assent and parental consent.

The study’s eligibility criteria included (1) self-identifying as Black/African American; (2) being between 14 and 24 years old; (3) residing in one of three Midwestern cities; (4) being assigned male at birth; (5) being fluent in English; (6) currently identifying as male; and (7) reporting sexual contact (oral, anal, or otherwise) with a male in the past year. For this study, responses from youth 14–17 years were only examined. Respondents who did not meet the inclusion criteria were excluded from the survey. To ensure all questions were answered, the Qualtrics forced-response option was used. Participants who completed the 20-min survey and provided an email address received a $35 electronic Visa gift card.

The research team verified respondents’ IP addresses were located within the United States. Data integrity was further safeguarded by restricting each respondent to only one completion of the eligibility and survey questions. A speeding check was implemented to exclude participants whose completion time was less than one-third of the median survey duration. To ensure data quality, avoid duplicate submissions, and minimize the likelihood of bot activity, the survey employed Qualtrics survey protection measures. These measures included browser cookies placed upon survey submission, reCAPTCHA verification through preliminary questions requiring respondents to identify specific items in pictures or replicate a series of letters, and bot detection via a Qualtrics survey question that provided a reCAPTCHA score indicating the likelihood of a respondent being a bot.

Upon clicking the survey link, participants were presented with an informed consent form and a screening tool to determine their eligibility for the study. Eligible participants were asked a series of questions about demographics, developmental assets (e.g., academic engagement), and suicidal behaviors. Participants recruited through social media completed the survey on personal computers or smartphones while those recruited through community-based organizations used the organization’s devices.

### Measures

#### Suicidality

Suicidal behaviors were assessed using a self-report adaptation of the Columbia–Suicide Severity Rating Scale (C-SSRS), which has been psychometrically supported for self-report use in community and online samples [14, 15]. Although the C-SSRS was originally developed as a clinician-administered interview encompassing multiple domains of suicidal ideation and behavior, the present study focused specifically on the suicidal behaviors subscale, consistent with prior research examining behavioral indicators of suicide risk in nonclinical samples [6, 12]. This subscale includes four dichotomous (Yes/No) items assessing the presence or absence of suicidal behaviors, including actual, interrupted, and aborted suicide attempts, as well as preparatory behaviors (e.g., writing a suicide note), assessed across the participant’s lifetime and within the past three months. Responses were summed to create a total suicidal behavior score, with possible values ranging from 0 to 4, where higher scores indicated endorsement of a greater number of suicidal behaviors. In addition, the number of suicide attempts was documented separately as a continuous variable. The subscale demonstrated strong internal consistency in this sample (Cronbach’s α = 0.87). To ensure participant safety, the survey concluded with a list of mental health and crisis resources, including the 988 Suicide and Crisis Lifeline, along with additional community-based supports.

#### Anticipated discrimination

Anticipated discrimination was measured using a nine-item subscale drawn from a multidimensional discrimination battery assessing intersectional stigma experiences [16]. This subscale captures participants’ expectations of experiencing unfair treatment in the future based on their social identities (e.g., race, gender, or sexual orientation). Participants rated the likelihood of each discriminatory event using a Likert-type scale ranging from 1 (very unlikely) to 5 (very likely), with higher scores reflecting greater anticipated discrimination. Example items include expectations of being disrespected, ignored, or stereotyped by others because of one’s identity. Item responses were averaged to create a composite score. Internal consistency reliability for the scale was excellent in the present sample (α = 0.92). Only the anticipated discrimination subscale was included in the current analyses, as it aligns with the study’s focus on the psychological burden of expected discriminatory treatment and its association with suicidal behaviors.

#### Developmental assets

Developmental assets were assessed with three internal asset subscales derived from previously validated measures with established reliability and construct validity [13, 27–31]. These measures have been widely used in investigating youth well-being and thus provide a robust foundation for assessing developmental assets across diverse populations.

#### Internal assets

*Positive identity* was assessed using a six-item scale with two subcategories: hope (three items) and self-esteem (three items). Each item was rated on a 5-point Likert scale, ranging from 1 (*Strongly disagree*) to 5 (*Strongly agree*), with higher scores indicating a more positive identity. The scale demonstrated adequate internal consistency (α = 0.71).

*Positive values* were assessed using a 10-item scale with four subscales that measured caring (three items), social justice (two items), integrity (two items), and responsibility (three items). Participants rated their level of agreement with each item on a 5-point Likert scale ranging from 1 (*Strongly disagree*) to 5 (*Strongly agree*). The combined 10-item scale demonstrated strong internal consistency (α = 0.89).

*Social competencies* were assessed via eight items across two domains: social-emotional skills (four items) and planning/decision-making skills (four items). Items were rated on a 5-point Likert scale ranging from 1 (*Strongly disagree*) to 5 (*Strongly agree*), with participants indicating their level of agreement with each item, including “Being good at making and keeping friends.” The scale demonstrated strong internal consistency (α = 0.88).

#### Statistical analysis plan

All analyses were conducted using STATA 18 for descriptive and preliminary statistics and M-plus 8.11 for multivariate modeling. Prior to analyses, data were examined for univariate outliers, normality, and multicollinearity. Overall missingness across all study variables was minimal (< 2%) and no single variable exceeded 2% missing data. Given the low level of missingness and the cross-sectional design, missing data were handled using complete-case estimation, which is unlikely to bias parameter estimates.

Descriptive statistics were computed for all study variables, including anticipated discrimination, suicidal behaviors, and the developmental asset subscales. Bivariate correlations were examined to assess zero-order associations among constructs and to evaluate conceptual distinctions among asset indicators. Reliability statistics (Cronbach’s alpha) were calculated for all multi-item scales.

To examine the association between anticipated discrimination and suicidal behaviors, linear regression models were estimated, given that suicidal behaviors were operationalized as a continuous outcome. All models controlled for age, education, income, and relationship status. Model fit was evaluated using R² values, and effect sizes were expressed as unstandardized regression coefficients (b) with corresponding standard errors (SE) and *p* values.

Moderation analyses (Table 3) tested whether developmental assets moderated the association between anticipated discrimination and suicidal behaviors. Mean-centered constituent variables were used to compute interaction terms (e.g., anticipated discrimination × positive identity) to reduce multicollinearity. Separate moderation models were estimated for each developmental asset, followed by a final model including all significant interaction terms. Significant interactions were probed using simple slopes analyses to estimate the association between anticipated discrimination and suicidal behaviors at low (− 1 SD), mean, and high (+ 1 SD) levels of the moderator. A developmental asset was interpreted as buffering when the association between anticipated discrimination and suicidal behaviors was weaker at higher levels of the asset. Multicollinearity diagnostics (variance inflation factors < 5) were used to confirm model stability. Results were compared for convergence and consistency across specifications.

## Results

### Descriptive statistics and correlations

Descriptive statistics and bivariate correlations for all study variables are presented in Table 1. The final analytic sample included *N* = 384 adolescents. Participants reported a mean suicidal behavior score of 7.06 (SD = 2.08) on the suicidal behaviors index (possible range: 0–12), indicating that, on average, youth endorsed multiple suicidal behaviors. Anticipated discrimination scores were relatively low (M = 3.00, SD = 2.76; range: 1–5). Developmental assets demonstrated moderate to high average levels, including positive identity (M = 3.31, SD = 0.80; range: 1–5), positive values (M = 3.88, SD = 0.85), and social competencies (M = 3.83, SD = 0.84).

**Table 1:** Descriptive Statistics and Bivariate Correlations Among Suicidal Behaviors, Anticipated Discrimination, and Developmental Assets

Variable	Mean	Standard deviation	1	2	3	4	5
1. Suicidal behaviors (C-SSRS)	7.06	2.08	–				
2. Anticipated discrimination	3.00	2.76	0.35**	–			
3. Positive identity	3.31	0.80	– 0.40**	– 0.28**	–		
4. Positive values	3.88	0.85	– 0.33**	– 0.25**	0.52**	–	
5. Social competencies	3.83	0.84	– 0.22**	– 0.18*	0.41**	0.44**	–

Range = Suicidal behaviors (0–10); Anticipated discrimination (1–5); Positive identity (1–5); Positive values (1–5); Social competencies (1–5). *p* < 00.05*. *p* < 0.01***

As expected, anticipated discrimination was positively associated with suicidal behaviors, *r* (382) = 0.35, *p* < 0.01, indicating that higher levels of anticipated discrimination corresponded with greater suicidal behaviors. All developmental asset variables were negatively associated with suicidal behaviors, ranging from *r* = – 0.22 to – 0.40 (all *p* < 0.05), suggesting that youth with stronger internal assets reported fewer suicidal behaviors. Developmental assets were moderately intercorrelated: positive identity was significantly associated with positive values (*r* = 0.52, *p* < 0.01) and social competencies (*r* = 0.41, *p* < 0.01). Positive values and social competencies were also positively associated with each other (*r* = 0.44, *p* < 0.01). Anticipated discrimination showed small to moderate negative correlations with all internal assets, ranging from *r* = – 0.18 to *r* = – 0.28 (*p* < 0.05). No correlation exceeded 0.70, indicating that multicollinearity was unlikely to cause a concern for subsequent multivariate analyses (See Table 1).

### Main effects

As hypothesized, anticipated discrimination was significantly and positively associated with suicidal behaviors (see Table 2). In the main effects model, higher levels of anticipated discrimination predicted greater suicidal behaviors (b = 0.53, SE = 0.07, *p* < 0.001, β = 0.27). Consistent with Hypothesis 2, higher levels of positive identity (b = − 0.57, SE = 0.10, *p* < 0.001, β = − 0.22) and social competencies (b = − 0.72, SE = 0.14, *p* < 0.001, β = − 0.29) were each associated with lower suicidal behaviors. Positive values were not significantly associated with suicidal behaviors (b = 0.10, SE = 0.13, *p* = 0.472, β = 0.04).

**Table 2:** Main effects of anticipated discrimination and internal assets on suicidal behaviors (*N* = 384)

Predictor	b	SE	t	*p*	β
Anticipated discrimination	0.53	0.07	7.05	< 0.001	0.27
Social competencies	− 0.72	0.14	−5.12	< 0.001	− 0.29
Positive values	0.10	0.13	0.72	0.472	0.04
Positive identity	− 0.57	0.10	−5.52	< 0.001	−0.22
Intercept	9.61	0.58	16.57	< 0.001	–
Model fit: R² = 0.27; Adjusted R² = 0.27; F(4, 378) = 49.69, *p* < 0.001; Root MSE = 1.69					

b = unstandardized regression coefficient; SE = standard error; β = standardized coefficient. Suicidal behaviors were modeled as a continuous outcome. All predictors were entered simultaneously. Higher scores indicate greater levels of each construct

### Moderation effects

#### Social competencies

**Fig. 2:**
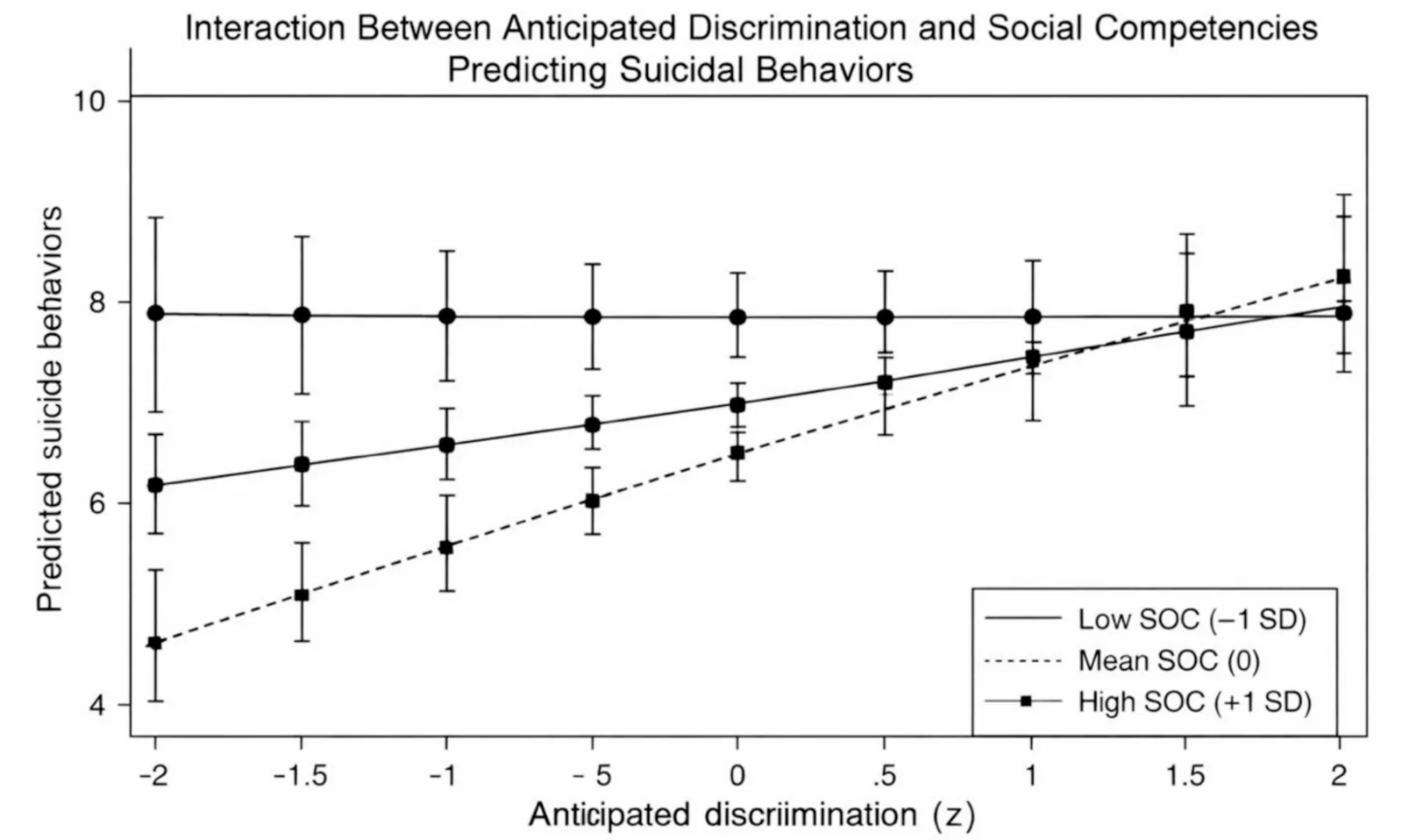
Interaction between anticipated discrimination and social competencies predicting suicidal behaviors. Predicted suicidal behavior scores are plotted as a function of anticipated discrimination (z-scored) at low (−1 SD), mean, and high (+1 SD) levels of social competencies. Solid, dashed, and dotted lines distinguish levels of social competencies in the black-and-white figure. Error bars represent 95% confidence intervals. Although social competencies were associated with lower suicidal behaviors overall, higher social competencies amplified the positive association between anticipated discrimination and suicidal behaviors

The interaction between anticipated discrimination and social competencies was statistically significant (b = 0.49, SE = 0.20, *p* = 0.015). Simple slopes analyses indicated that the association between anticipated discrimination and suicidal behaviors was strongest among individuals with high levels of social competencies (+ 1 SD: b = 0.94, *p* < 0.001), moderate at the mean level (b = 0.46, *p* = 0.001), and nonsignificant among those with low levels (− 1 SD: b = − 0.03, *p* = 0.911; Fig. 2; Table 3). This pattern suggests that, contrary to expectations, higher social competencies may heighten vulnerability to the psychological effects of anticipated discrimination, potentially reflecting increased social awareness, vigilance, or exposure to stigmatizing interactions.

**Table 3:** Moderation of the association between anticipated discrimination and suicidal behaviors (*N* = 384)

Predictor	b	SE	t	*p*	95% CI [LL, UL]
Model 1: anticipated discrimination × social competencies					
Anticipated discrimination (AD)	0.46	0.13	3.44	< 0.001	[0.20, 0.72]
Social competencies	− 0.78	0.17	− 4.44	< 0.001	[− 1.12, − 0.43]
AD × Social competencies	0.49	0.20	2.44	0.015	[0.09, 0.88]
Model 2: anticipated discrimination × positive values					
Anticipated discrimination	0.46	0.13	3.46	< 0.001	[0.20, 0.72]
Positive values	− 0.48	0.10	− 4.92	< 0.001	[− 0.68, − 0.29]
AD × Positive values	0.05	0.13	0.42	0.675	[− 0.20, 0.30]
Model 3: anticipated discrimination × positive identity					
Anticipated discrimination	0.71	0.09	7.76	< 0.001	[0.53, 0.89]
Positive identity	− 0.58	0.09	− 6.27	< 0.001	[− 0.76, − 0.40]
AD × Positive identity	− 0.21	0.09	− 2.30	0.022	[− 0.39, − 0.03]

#### Positive identity

**Fig. 3:**
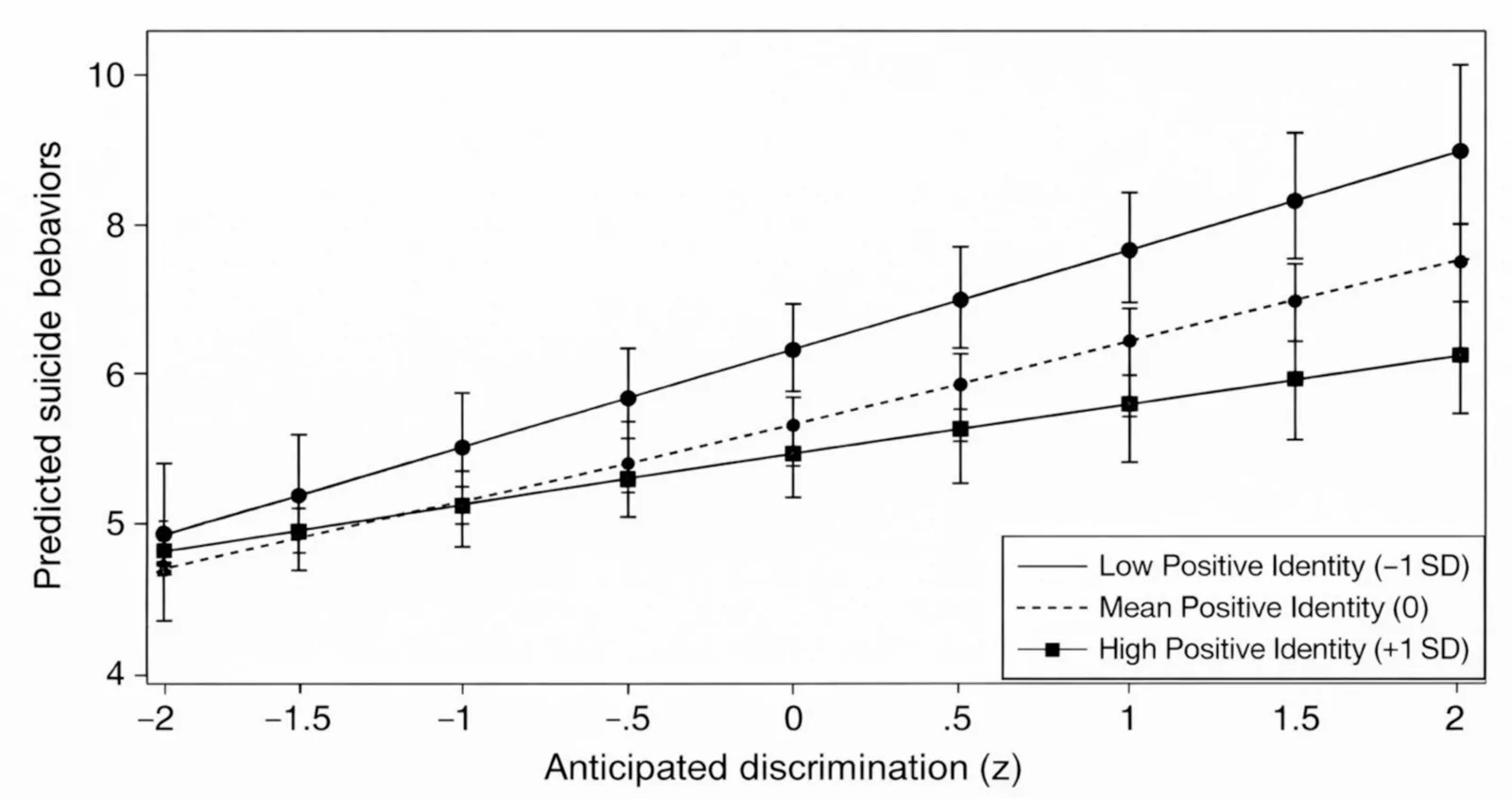
Interaction between anticipated discrimination and positive identity predicting suicidal behaviors. Predicted suicidal behavior scores are plotted as a function of anticipated discrimination (z-scored) at low (−1 SD), mean (0), and high (+1 SD) levels of positive identity. Solid, dashed, and dotted lines distinguish levels of positive identity in the black-and-white figure. Error bars represent 95% confidence intervals. The association between anticipated discrimination and suicidal behaviors was strongest at low levels of positive identity and attenuated at high levels, indicating a buffering effect

Consistent with our hypothesis, the interaction between anticipated discrimination and positive identity was statistically significant (b = − 0.23, SE = 0.09, *p* = 0.011). Simple slopes analyses indicated that the association between anticipated discrimination and suicidal behaviors was strongest among individuals with low levels of positive identity (− 1 SD: b = 0.69, *p* < 0.001), moderate at the mean level (0 SD: b = 0.46, *p* = 0.001), and nonsignificant among those with high levels of positive identity (+ 1 SD: b = 0.22, *p* = 0.160; Fig. 3; Table 3). This pattern indicates that a strong and coherent sense of identity may mitigate the psychological toll of anticipated discrimination on suicidal behaviors.

As shown in Table 4, the conditional effects of anticipated discrimination on suicidal behaviors varied across levels of social competencies and positive identity. For social competencies, anticipated discrimination was not significantly associated with suicidal behaviors at low levels of social competencies, *b* = − 0.03, *SE* = 0.27, *t* = − 0.11, *p* = 0.911, 95% CI [− 0.55, 0.49]. However, anticipated discrimination was positively associated with suicidal behaviors at mean levels, *b* = 0.46, *SE* = 0.13, *t* = 3.44, *p* < 0.001, 95% CI [0.20, 0.72], and high levels of social competencies, *b* = 0.94, *SE* = 0.21, *t* = 4.52, *p* < 0.001, 95% CI [0.53, 1.35]. For positive identity, anticipated discrimination was positively associated with suicidal behaviors at low levels of positive identity, *b* = 0.69, *SE* = 0.16, *t* = 4.27, *p* < 0.001, 95% CI [0.37, 1.01], and mean levels of positive identity, *b* = 0.46, *SE* = 0.13, *t* = 3.47, *p* < 0.001, 95% CI [0.20, 0.72]. However, the association was not significant at high levels of positive identity, *b* = 0.22, *SE* = 0.16, *t* = 1.41, *p* = 0.160, 95% CI [− 0.09, 0.54].

**Table 4:** Conditional effects of anticipated discrimination on suicidal behaviors at low, mean, and high levels of each moderator (*N* = 384)

Moderator	Moderator level	b	SE	t	*p*	95% CI [LL, UL]
Social competencies	*−1 SD*	− 0.03	0.27	− 0.11	0.911	[− 0.55, 0.49]
	0 (*M*)	0.46	0.13	3.44	< 0.001	[0.20, 0.72]
	*+ 1 SD*	0.94	0.21	4.52	< 0.001	[0.53, 1.35]
Positive identity	*−1 SD*	0.69	0.16	4.27	< 0.001	[0.37, 1.01]
	0 (*M*)	0.46	0.13	3.47	< 0.001	[0.20, 0.72]
	+ 1 *SD*	0.22	0.16	1.41	0.160	[− 0.09, 0.54]

## Discussion

This study is among the first to examine the association between anticipated discrimination and suicidal behaviors among Black gay and bisexual male adolescents and to test whether internal developmental assets buffer (or exacerbate) this association. Consistent with minority stress theory and prior evidence documenting disproportionate suicide risk among LGBTQ+ youth, anticipated discrimination emerged as a robust predictor of suicidal behaviors [2–4]. This finding extends previous work demonstrating that racism, homophobia, and HIV-related stigma contribute to elevated psychological distress and suicidality among BGBMA [7, 8, 12, 19, 21]. Together, these findings provided the basis for examining whether internal developmental assets functioned as protective factors that could alter the association between anticipated discrimination and suicidal behaviors.

Positive identity emerged as a significant protective factor, both as a main effect and as a moderator. Higher levels of positive identity were associated with lower suicidal behaviors, and positive identity attenuated the association between anticipated discrimination and suicidality. This pattern is consistent with the Developmental Assets Framework, which emphasizes the role of internal assets such as positive identity, positive values, and social competencies in promoting resilience and psychological well-being [27, 29]. These findings suggest that a strong, affirming sense of self may help Black sexual minority adolescents resist the internalization of discriminatory expectations and maintain psychological stability in the face of anticipated stigma. This interpretation aligns with prior research demonstrating that positive identity reduces depression and suicidality among sexual minority youth [11, 31] and extends emerging work highlighting the protective role of internal assets among BGBMA [6].

In contrast, social competencies demonstrated a different pattern. Although higher social competencies were associated with lower overall levels of suicidal behaviors, they amplified the association between anticipated discrimination and suicidality at higher levels. This finding suggests that socially skilled youth are not uniformly protected from the effects of anticipated discrimination. One possible explanation is that youth with higher social competencies may exhibit heightened social awareness or vigilance, increasing their sensitivity to cues of rejection or anticipated mistreatment. This pattern may also be understood through the Rejection Sensitivity Model, which posits that individuals who are more attuned to interpersonal cues may be more likely to anticipate and perceive rejection, thereby intensifying emotional responses to potential stigma [34]. Among sexual minority adolescents, heightened rejection sensitivity has been linked to increased distress and poorer mental health outcomes, particularly in contexts where discrimination is expected [35]. Consistent with this interpretation, prior work suggests that greater social attunement can intensify emotional labor, stress appraisal, and awareness of stigma [25]. Additionally, greater social engagement may increase exposure to environments where discrimination is anticipated or experienced. It is also possible that socially competent youth feel increased pressure to effectively navigate stigmatizing interactions, which may heighten the psychological burden of anticipated discrimination. Although rejection sensitivity was not directly assessed in the present study, future research should incorporate validated measures to more precisely test this mechanism. Together, these findings highlight that developmental assets may function in complex, context-dependent ways and underscore the importance of examining how specific assets interact with minority stress processes.

The nonsignificant moderation for positive values is notable. Although descriptive patterns aligned with a buffering hypothesis, positive values did not significantly attenuate the discrimination-suicidality link. This suggests that moral orientations (e.g., social justice, responsibility, or caring) may be less protective against discrimination-induced distress than assets tied to personal identity and social-emotional functioning. Given the mixed evidence regarding positive values in other populations, future research should examine how these assets operate in Black sexual minority adolescents’ day-to-day experiences, including whether certain values may activate stress responses or moral injury when confronted with identity-based unfairness.

Taken together, these findings highlight the value of integrating minority stress theory with the Developmental Assets Framework to understand suicide risk among BGBMA. Guided by minority stress theory, the study underscores how anticipatory processes including vigilance and expectations of rejection may function as meaningful drivers of suicide risk during adolescence, a developmental period marked by identity formation and heightened sensitivity to social evaluation. At the same time, the findings demonstrate that internal assets, including positive identity and social competencies, shape how youth experience and respond to these stressors. Importantly, these assets did not operate uniformly: positive identity appeared protective, whereas social competencies intensified the association between anticipated discrimination and suicidal behaviors. This pattern underscores that suicide risk among BGBMA is not only a function of exposure to stigma, but also of how developmental strengths interact with anticipatory stress processes in complex and context-dependent ways,

### Theoretical implications

The current study advances theory in several important ways. First, the findings extend minority stress theory by demonstrating that anticipated discrimination—a future-oriented expectation rather than an enacted experience—is strongly associated with suicidal behaviors among BGBMA. These findings extend minority stress theory by highlighting anticipatory processes as a critical pathway through which stigma-related stress may shape mental health and suicide risk among BGBMA [13–15, 17]. By centering anticipated discrimination, the present findings suggest that expectations of future mistreatment may intensify psychological burden through heightened vigilance, anxiety, hopelessness, and internalized stigma [13–16]. The salience of anticipated discrimination during adolescence and young adulthood underscores how stigma-related vigilance may become internalized early in the life course, shaping emerging mental health trajectories.

Second, this study integrates the Developmental Assets Framework with minority stress and intersectionality perspectives, illustrating that internal assets operate differently across stressor types and asset domains. This integration responds to long-standing calls to adapt strength-based developmental models for youth whose environments are marked by racism, heterosexism, and HIV-related stigma [8, 27, 28]. At the same time, these findings suggest that protective assets do not operate uniformly; rather, their buffering effects may depend on the specific asset, the nature of anticipated discrimination, and the suicide-related outcome examined [13, 14]. The observed patterns—particularly the differential moderating roles of positive identity versus social competencies—suggest that developmental assets may function differently depending on how youth perceive and anticipate discrimination. These findings extend both frameworks by demonstrating that protective factors may vary in effectiveness depending on the nature of the stressor and the sociocultural context in which it is embedded.

Third, the significant buffering role of positive identity emphasizes the central importance of identity-related strengths for Black sexual minority youth, who must integrate racial and sexual identities in contexts where both are frequently stigmatized [8, 19]. This finding aligns with emerging work within minority stress theory highlighting resilience processes and identity affirmation as critical mechanisms that can disrupt the internalization of stigma [14, 15]. Positive identity may function as a core developmental asset that mitigates the psychological impact of anticipated discrimination by reinforcing self-worth and counteracting negative expectations. In contrast, the lack of buffering effects for positive values and the unexpected amplification associated with social competencies point to the need for more nuanced theoretical models that account for the complex ways in which assets shape stress reactivity, vigilance, and coping. Together, these contributions underscore the importance of theory that is culturally grounded, developmentally informed, and responsive to the intersecting forms of stigma experienced by BGBMA.

### Clinical implications

Findings from this study highlight several important implications for clinical practice with BGBMA. First, youth who anticipate future discrimination exhibit significantly elevated suicidal behaviors, underscoring the need for clinicians to assess anticipated discrimination—not solely past victimization—during suicide risk evaluations. Clinicians may consider incorporating brief, developmentally appropriate questions into standard assessments, such as asking youth whether they expect to be treated unfairly in school, healthcare, community, or family settings due to their race, sexual orientation, or other identities, and how often they worry about or prepare for such experiences. Assessing anticipatory vigilance, expectations of rejection, and perceived likelihood of discrimination may help identify youth whose stress exposure is not yet observable through enacted events but is nonetheless clinically significant.

Second, positive identity emerged as a robust protective factor, suggesting that interventions that affirm racial, sexual, and intersecting identities may play a critical role in mitigating the psychological consequences of discrimination-related stress. Strengths-based approaches that promote self-esteem, hope, and pride in one’s identities may be especially beneficial for BGBMA navigating stigmatizing environments. In addition to risk assessment, clinicians should incorporate asset-based assessments that evaluate internal strengths (e.g., identity, coping, social skills) to more fully understand both vulnerability and resilience in this population.

Although social competencies were associated with fewer overall suicidal behaviors, they unexpectedly intensified the relationship between anticipated discrimination and suicidality at higher levels. This finding cautions clinicians against assuming that socially skilled or “high-functioning” youth are at low risk. Instead, youth with strong social competencies may benefit from additional support around managing stigma-related stress, emotional boundaries, and heightened social vigilance. Clinicians may also consider assessing how youth interpret social interactions and whether heightened awareness of potential discrimination contributes to distress.

More broadly, suicide prevention efforts for Black sexual minority youth should integrate the Developmental Assets Framework with minority stress and intersectionality perspectives. Programs that strengthen internal assets must be paired with interventions that directly address racism, heterosexism, and HIV-related stigma across school, family, healthcare, and community contexts. Finally, family-focused and community-based approaches that promote family acceptance, open communication, and culturally grounded support remain essential. Given the central role of family and social environments in shaping the development of internal assets and exposure to stigma, enhancing these support systems is critical for reducing suicide risk among BGBMA.

### Limitations and future directions

Several limitations warrant consideration. The cross-sectional design precludes causal inference and limits conclusions about temporal ordering; longitudinal studies are needed to examine how anticipated discrimination shapes trajectories of suicidality. Reliance on self-report measures may introduce reporting biases, including social desirability and recall error. Although the sample spanned three Midwestern cities, generalizability to youth in rural areas and other regions remains uncertain. The anticipated discrimination measure, while demonstrating strong internal consistency, was derived from a broader discrimination battery and requires further validation for Black sexual minority adolescents. Finally, although guided by the Developmental Assets Framework, this study focused exclusively on internal assets and did not include external assets (e.g., family support, school climate, community connectedness). The omission of these contextual factors may limit a full ecological understanding of risk and resilience and may have influenced the magnitude and interpretation of observed associations. Future research should integrate multilevel assets to more comprehensively test developmental and minority stress processes. Longitudinal, multilevel investigations are needed to clarify underlying mechanisms and translate these findings into targeted, culturally responsive suicide prevention strategies for Black sexual minority adolescents.

## Conclusion

This study highlights anticipated discrimination as a critical and understudied pathway linked to suicidal behaviors among Black gay and bisexual male adolescents. By integrating minority stress theory with the Developmental Assets Framework, the findings demonstrate that internal developmental assets operate in nuanced, context-dependent ways, with positive identity emerging as a key protective factor that attenuates the impact of anticipated discrimination. At the same time, the differential role of social competencies underscores the complexity of how strengths interact with stigma-related stress. Together, these findings emphasize the need for developmentally and culturally grounded suicide prevention efforts that both strengthen internal assets and directly address the anticipatory and structural dimensions of discrimination shaping the lives of Black sexual minority youth.

## Data Availability

Data that support the findings of this study are available on request under a license agreement. Written applications can be made to the author corresponding author [(boyd.465@osu.edu) (mailto: boyd.465@osu.edu)].
